# Genetic dissection of agronomic and quality traits based on association mapping and genomic selection approaches in durum wheat grown in Southern Spain

**DOI:** 10.1371/journal.pone.0211718

**Published:** 2019-02-27

**Authors:** Rosa Mérida-García, Guozheng Liu, Sang He, Victoria Gonzalez-Dugo, Gabriel Dorado, Sergio Gálvez, Ignacio Solís, Pablo J. Zarco-Tejada, Jochen C. Reif, Pilar Hernandez

**Affiliations:** 1 Instituto de Agricultura Sostenible (IAS) Consejo Superior de Investigaciones Científicas (CSIC), Alameda del Obispo s/n, Córdoba, Spain; 2 Department of Breeding Research, Leibniz Institute of Plant Genetics and Crop Plant Research (IPK) Gatersleben, Corrensstraße 3, Stadt Seeland, Germany; 3 Departamento de Bioquímica y Biología Molecular, Campus Rabanales C6-1-E17, Campus de Excelencia Internacional Agroalimentario (ceiA3), Universidad de Córdoba, Córdoba, Spain; 4 Universidad de Málaga, Andalucía Tech, ETSI Informática, Campus de Teatinos s/n, Málaga, Spain; 5 ETSIA (University of Seville), Ctra de Utrera km1, Seville, Spain; Università Politecnica delle Marche, ITALY

## Abstract

Climatic conditions affect the growth, development and final crop production. As wheat is of paramount importance as a staple crop in the human diet, there is a growing need to study its abiotic stress adaptation through the performance of key breeding traits. New and complementary approaches, such as genome-wide association studies (GWAS) and genomic selection (GS), are used for the dissection of different agronomic traits. The present study focused on the dissection of agronomic and quality traits of interest (initial agronomic score, yield, gluten index, sedimentation index, specific weight, whole grain protein and yellow colour) assessed in a panel of 179 durum wheat lines (*Triticum durum* Desf.), grown under rainfed conditions in different Mediterranean environments in Southern Spain (Andalusia). The findings show a total of 37 marker-trait associations (MTAs) which affect phenotype expression for three quality traits (specific weight, gluten and sedimentation indexes). MTAs could be mapped on the A and B durum wheat subgenomes (on chromosomes 1A, 1B, 2A, 2B and 3A) through the recently available bread wheat reference assembly (IWGSC RefSeqv1). Two of the MTAs found for quality traits (gluten index and SDS) corresponded to the known *Glu-B1* and *Glu-A1* loci, for which candidate genes corresponding to high molecular weight glutenin subunits could be located. The GS prediction ability values obtained from the breeding materials analyzed showed promising results for traits as grain protein content, sedimentation and gluten indexes, which can be used in plant breeding programs.

## Introduction

Durum wheat (*Triticum durum*) is one of the most important crops in the Mediterranean diet. It is mainly grown in the Mediterranean basin (Italy, Turkey, Algeria and Spain, providing 50% of the world's production [1, 2]) and North America (Canada, Mexico and USA). The genetic dissection of agronomic and quality traits is essential for durum breeding programs. The identification of QTLs related to quality and yield is important as an entry point for marker assisted selection (MAS) [3]. Association mapping (AM) is an integrated analysis to determine genotype-phenotype correlations in a germplasm collection [4] based on the linkage disequilibrium (LD). AM mapping resolution depends on the number and density of markers [5], on the ability to correctly measure the target trait and the traits of the population under study, and on an efficient field design [6]. It has been used to dissect several agronomic traits of great importance in bread and durum wheat, such as yield or yield-related traits [7, 8], quality [9, 10], biotic stress resistance [11, 12] and abiotic stress tolerance [13, 14].

While MAS uses markers which are significantly linked to qualitative traits, and is integrated with traditional phenotypic selection and long selection cycles [15], genomic selection (GS) appears as an alternative approach which considers complex quantitative traits using genome-wide markers [15]. GS estimates simultaneously all the loci effects across the complete genome to compute genomic values (GEBVs) of lines for selection by using the sum of the marker effects which they contain [16], and its potential in plant breeding has already been proved [15, 17–20]. It has been suggested as a plant breeding methodology that accelerates the breeding cycle and provides a rapid selection of better genotypes for a low cost [15, 21, 22].

The application of GS in plant breeding programmes is possible due to the availability of high-throughput molecular markers, which cover the entire genome and facilitate trait value prediction [21, 23, 24]. Experimental studies based on multi-environment CIMMYT (International Maize and Wheat Improvement Center) wheat and maize trials showed that genomic selection models present a considerable prediction ability for genetic values of complex traits such as grain yield or adaptability to different stresses under markedly different conditions [3, 24, 25].

Durum wheat is well-adapted to semi-arid and arid environments as the Mediterranean [26], despite this is an heterogeneous region with a broad range of soil fertility levels, temperatures and rainfall. In Mediterranean agricultural environments, high quality durum wheat is produced [27], mainly under rainfed conditions. The main abiotic factors limiting the crop’s growth and final yield are drought and heat stresses [27–29]. Mediterranean environments are characterized by high water deficit and high temperatures during anthesis and grain filling stages [27, 29]. Low rainfall and its erratic distribution, mainly winter-dominated rainfalls, account for approximately 75% of variations in final yield [30]. These environmental constraints significantly influence the expression of many important agronomic traits such as grain yield [29, 31], sedimentation volume and grain protein content [32], which are main targets of durum wheat breeding programmes.

Several AM and GS analyses of yield and quality traits in durum wheat, were performed in limiting environments [8, 13, 33–35]. Maccaferri et al. [13], analyzed durum elite lines in different Mediterranean countries, Mexico and USA, using SSR markers and a broad range of soil moisture. Recently, Sukumaran et al. [8, 33] assessed CIMMYT durum wheats grown under three different conditions (yield potential, drought and heat stresses) using DArTseq markers.

The present study was carried out in different areas in Southern Spain (Andalusia), which produces the 70% of the Spanish durum wheat production (http://www.aetc.es/). This cropping area presents different macro-environments, which differ in temperature and quantity of precipitations. These unpredictable conditions result in important abiotic stresses, mainly drought and/or heat stresses, which strongly affect the final phenological stages, such as anthesis and grain filling [31]. These erratic variations in rainfall and extreme temperatures in Southern Spain strongly influence important traits as final yield, protein content and quality indices [36]. To dissect the genetic basis of quality and yield in these particular environments, a set of CIMMYT elite lines and local varieties presenting a lack of genetic structure was tested, highlighting the importance of testing the previously selected genotypes in additional local environments. Genome-wide markers were used to analyse and compare the potential and limits of the MAS and GS approaches to improve agronomic and quality traits in durum wheat grown under rainfed Mediterranean agro-climatic conditions.

## Material and methods

### Plant material and field trials

A panel of 160 experimental CIMMYT elite durum wheat breeding lines and 19 durum wheat varieties (S1 Table) were grown in a Mediterranean area under rainfed conditions, throughout three cropping seasons (from 2013 to 2015). All 179 genotypes were tested in field trials in two locations in the provinces of Seville and Huelva (37° 32' 18" N, 5° 6' 17" O and 37° 27' 28" N, 6° 21' 52" O). The 19 released varieties were grown additionally at three more locations: two in the province of Cadiz (36° 16' 8" N, 6° 4' 30" O and 36° 42' 12" N, 6° 10' 8" O) and one in the province of Cordoba (37° 47' 21" N, 4° 36' 28" O). These five locations were diverse in terms of rainfall, temperatures, altitude, soil type and texture (S2 and S3 Tables) and represent the two agro-climatic cereal-growing environments present in Southern Spain. Based on the method proposed by Papadakis [37], the sites in the province of Cadiz are classified as maritime Mediterranean environments, with high environmental humidity values; while the sites in the provinces of Seville, Huelva and Cordoba are climatically classified as subtropical Mediterranean environments, characterised by mild, wet winters with irregular precipitations and hot, dry summers. The experimental lines assessed were elite genotypes, pre-selected by CIMMYT based on their yield stability across environments and high quality. The aim of the breeding strategy was the adaptation to Southern Spain agroclimatic conditions.

The experimental design consisted of one randomized complete block with three replications of the varieties at the five locations indicated above; and a randomized complete block design with one plot per experimental line at two of those sites (Seville and Huelva). The trials were planted in 7.2m^2^ plots, using a sowing density of 360 seeds/m^2^ for Seville, Huelva and one of the sites of Cadiz, while in Cordoba and the second site in Cadiz, the seed density was adjusted according to the worst estimated nascence of seeds (396 seeds/m^2^) due to the high clay soil content. Fields were managed following the standard agricultural practices in each location (S3 Table) and all trials were performed under non-irrigated conditions.

Seven agronomic traits were evaluated at different stages of development: initial agronomic score (IAS), specific weight (g, SW), gluten index (%, GI), sedimentation index (cm^3^, SDS), whole grain protein (%, WGP), yellow colour (YC) and grain yield (kg/ha, YIELD). IAS was the only trait which was visually assessed at the field trials, and consists of evaluating the seedling vigour and amount of soil covered as a value, that for elite material falls within a typical 5–10 range (<5 = very poor; 5 = poor, 6 = fair, 7 = acceptable, 8 = good, 9 = very good and 10 = excellent). For quality assessment, SW and WGP were measured using Near-infrared spectroscopy (NIRs), following Williams and Norris [38]; SDS was evaluated by UNE 34903:2014 [39–41]; GI by ISO 21415:2016 [42]; and YC by using CEN/TS 15465:2008 [43–45].

There was no specific permission required for measuring data on the wheat farm trials. The on-farm field studies did not involve endangered or protected species.

### Phenotypic data analyses

Firstly, the correlations among the three replicates of the varieties in the two locations used for the experimental lines were analysed using the ‘cor.test’ function in R.

The adjusted entry means for each year for the association mapping study was estimated based on the following model:

$$p_{ikn}=\mu +g_{i}+l_{k}+{\left(gl\right)}_{ik}+\varepsilon_{ikn},$$

where *p*_*ikn*_ was the trait performance of the *i*^*th*^ genotype in the *n*^*th*^ replicate of the *k*^*th*^ location, *μ* was the intercept, *g*_*i*_ was the genetic effect of the *i*^*th*^ genotype, *l*_*k*_ was the effect of the *k*^*th*^ location, (*gl*)_*ik*_ was the genotype-by-location interaction effect of the *i*^*th*^ genotype in the *k*^*th*^ location, and *ε*_*ikn*_ was the corresponding residual. Only *μ* and *g*_*i*_ were treated as fixed effects.

The adjusted means of each genotype over the years was estimated with the following model:

$$p_{ij}=\mu +g_{i}+y_{j}+\varepsilon_{ij},$$

where *p*_*ij*_ was the trait performance of the *i*^*th*^ genotype in the *j*^*th*^ year, *μ* was the intercept, *g*_*i*_ was the genetic effect of the *i*^*th*^ genotype, *y*_*j*_ was the effect of the *j*^*th*^ year, and *ε*_*ij*_ was the corresponding residual. Only *μ* and *g*_*i*_ were treated as fixed effects. The adjusted means over the years were used to calculate the phenotypic correlation (Pearson correlation coefficient) across the traits.

To provide an overview of the different sources of the phenotypic variation for both experimental lines and released varieties and to estimate heritability, we fitted the following model:

$$p_{imn}=\mu +{\left(gt\right)}_{i}+{\left(gc\right)}_{i}+e_{m}+{\left(gte\right)}_{im}+\varepsilon_{imn},$$

where *p*_*imn*_ was the trait performance of the *i*^*th*^ genotype in the *n*^*th*^ replication of *m*^*th*^ environment (year-by-location combination), *μ* was the intercept, (*gt*)_*i*_ was the genetic effect of the *i*^*th*^ tester, (*gc*)_*i*_ was the genetic effect of the *i*^*th*^ candidate, *e*_*m*_ was the effect of the *m*^*th*^ environment, and *ε*_*imn*_ was the corresponding residual. Only *μ* was treated as a fixed effect. The variance components for experimental lines and durum wheat varieties were extracted separately by using the ‘dummies’ package in R. The significance of variance component estimates was tested by model comparison with likelihood ratio tests where the halved P values were used as an approximation [46]. Broad-sense heritability was estimated for released varieties as $h^{2}=\frac{\sigma_{gt}^{2}}{\sigma_{gt}^{2}+\frac{\sigma_{gte}^{2}}{Nr.Env}+\frac{\sigma_{\varepsilon }^{2}}{Nr.Env*Nr.Rep}}$. Broad-sense heritability was estimated for experimental lines as $h^{2}=\frac{\sigma_{gc}^{2}}{\sigma_{gc}^{2}+\frac{\sigma_{\varepsilon }^{2}}{Nr.Env}}$. Here $\sigma_{gt}^{2}$ and $\sigma_{gc}^{2}$ are the genotypic variance for testers and candidates, $\sigma_{gte}^{2}$ was variance of genotype-by-environment interaction of testers and $\sigma_{\varepsilon }^{2}$ was the variance of the residuals. *Nr*.*Env* and *Nr*.*Rep* represent the number of environments and number of replicates, respectively.

To extract the overall variance components for the tester population, we fitted the following model:

$$p_{imn}=\mu +g_{i}+e_{m}+\varepsilon_{imn},$$

where *p*_*imn*_ was the trait performance of the *i*^*th*^ genotype in the *n*^*th*^ replication of *m*^*th*^ environment (year-by-location combination), *μ* was the intercept, *g*_*i*_ was the genetic effect of the *i*^*th*^ genotype, *e*_*m*_ was the effect of the *m*^*th*^ environment and *ε*_*imn*_ was the corresponding residual. Only *μ* was treated as a fixed effect. Broad-sense heritability was estimated for released varieties as $h^{2}=\frac{\sigma_{g}^{2}}{\sigma_{g}^{2}+\frac{\sigma_{\varepsilon }^{2}}{Nr.Env*Nr.Rep}}$. The genetic variation extracted under this model was used in genomic prediction.

### Genotyping and population structure analyses

Plant tissue samples were obtained at the 4-leaf stage and the tissue was immediately frozen using dry ice. The DNA was isolated using approximately 100mg of frozen leaf and the DNeasy Plant Mini Kit from Qiagen, following the manufacturer’s instructions. The concentration and quality of the DNA samples were assessed by electrophoresis in a 0.8% agarose gel using lambda DNA as the standard. The absence of nucleases in the DNA samples was checked by performing an incubation at 37°C using a restriction enzyme (*Tru*1I) from ThermoFisher before the DartSeq analysis. The results were visualized by electrophoresis in a 0.8% agarose gel. DartSeq^TM^ genotyping and mapping of the corresponding markers of the wheat genome sequence from the International Wheat Genome Sequencing Consortium (IWGSC) was performed at Diversity Arrays (diversityarrays.com), as described by Sukumaran et al. [8].

All the markers with a minor allele frequency (MAF) below 5% were filtered out and a missing ratio over 5%. After quality control, 16,383 DArT and 5,649 single-nucleotide polymorphism (SNP) markers remained. The remaining missing values were imputed following He et al. [47]. The kindship matrices for the DArT and SNP markers were calculated based on Roger’s distances (S4 and S5 Tables). The correlation between the two kindship matrices was calculated using the ‘mantel’ function of the ‘vegan’ package in R.

The population structure was assessed applying principal coordinates analyses (PCoA) based on modified Rogers’ distances [48] using the “prcomp” function in R. The first and second principal coordinates were used to draw the two-dimensional space graph. In addition, a heatmap plot was drawn for the modified Roger’s distances in combination with cluster analysis by R function “uclust” using the “complete linkage” method. All further calculations were made using R.

### Genome-wide association analysis and linkage disequilibrium

The following mixed linear model was used for association mapping:

Y=Wa+Xβ+Ss+Zu+e,

where *Y* stands for the adjusted entry means of the genotypes per year, *a* is a vector of group effects, *β* is a vector of year effects, *s* is a vector of SNP effects, *u* is a vector of polygene background effects and *e* is a vector of residual effects. *W*, *X*, *S*, and *Z* are incidence matrices relating *Y* to *a*, *β*, *s*, and *u*, respectively. To check whether the population structure was adequately controlled by the model, a QQ-plot was drawn, based on the observed P-values and expected P-values of all markers. The significance of marker-trait associations was tested with the Wald F statistic. The false discovery rate (FDR) controlling procedure [49] was used to correct for multiple testing. After the correction, a value of 0.1 was set as threshold. The proportion of the phenotypic variance explained by a single QTL (R^2^) was estimated using analysis of variance (ANOVA) with QTLs reordered according to the P-values, and the effects of detected QTLs were estimated using a standard multiple regression approach. The genome-wide associations study (GWAS) was performed using the software ASREML-R. Associated DartSeq and SNP markers were blasted against the wheat reference assembly RefSeqv1 (IWGSC 2018) with no indels or mismatches allowed, using an ad hoc Java program, to confirm their physical mapping location on the A or B genomes. For candidate gene identification, the results were filtered selecting those hits with best *e-value* for each marker and the candidate genes were manually selected based on gene annotations. Differential gene expression analyses were carried out using RefSeqv1 gene models and two R libraries (Kallisto, version 0.43.0 and STAR DESeq2, version 1.14.1).

For linkage disequilibrium (LD), the algorithm R^2^ was used. This value was estimated between any pair of markers within one chromosome. To determine the genome-wide linkage disequilibrium, mapped SNP markers were used in the panel of 179 wheat lines. The calculations were made using Python to establish the average LD decay.

### Genome-wide prediction

Based on the adjusted entry means over the years, a ridge regression best linear unbiased prediction (RR-BLUP) was applied. Details of the implementation of the models have been described earlier [50]. Briefly, the general form of the models is defined as follows:

$$Y=1_{n}\mu +Z_{A}a+\varepsilon ,$$

where *Y* is the adjusted entry means over the years, 1_*n*_ is the vector of ones, *n* is the number of genotypes, *a* was the additive marker effect, *Z* is the design matrix for additive effects of the markers and *ε* is the residual.

The prediction ability, which was defined as the Pearson’s correlation coefficient between predicted values and adjusted entry means, was checked by five-fold cross-validation. 1000 cross-validation runs were performed and for each run, four fifths of the genotypes were randomly sampled as a training population to estimate marker effects, which were then used to predict the performance of the remaining genotypes. Genomic prediction was applied separately to SNP and DArT markers.

## Results

### Phenotypic data analysis

To verify the appropriateness of the assessed breeding trial design (which uses partly unreplicated trials for the experimental lines) for the subsequent statistical analyses, yield correlations were analysed among the three replicates of the varieties at the two sites, and found mean estimates of 0.70 (ranging from 0.42 to 0.97).

Variance components of the total samples are shown in Table 1. Descriptive statistics of each trait in each location with key quantiles are shown in S6 Table. For the experimental lines, the agronomic trait showing the highest heritability (h^2^) was specific weight (SW) with h^2^ = 0.71, followed by initial agronomic score (IAS) and whole grain protein (WGP) with h^2^ = 0.63 and h^2^ = 0.61, respectively. As expected, the h^2^ value for YIELD was low (h^2^ = 0.13). For released varieties, the traits with the highest heritability values were GI, IAS and SDS, with h^2^ = 0.88, 0.85 and 0.80, respectively. The heritability of WGP was also higher in the released varieties (h^2^ = 0.74) than in the experimental lines (h^2^ = 0.61). In contrast with the experimental lines, for released varieties the SW presented low heritability (h^2^ = 0.30), while the YIELD showed a high value (h^2^ = 0.85), probably as consequence of the reduced number of analysed varieties.

**Table 1:** Analysis of variance for the assessed traits.

	YIELD	WGP	SW	GI	IAS	SDS	YC
**σ**^**2**^_**g**_ ^a^	43165.69	0.17	0.90	95.54	0.03	21.84	7.53
**σ**^**2**^_**g-p**_ ^a^	1.94E-13	5.93E-06	0.31	5.70E-16	2.81E-12	4.43E-10	5.31E-05
**σ**^**2**^_**ge**_ ^a^	94915.76	0.32	24.12	NA	0.06	NA	NA
**σ**^**2**^_**ge_p**_ ^a^	5.39E-36	3.89E-05	2.06E-97	NA	8.93E-41	NA	NA
***Error*** ^a^	165311.91	0.76	1.68	158.62	0.08	63.18	47.54
**σ**^**2**^_**g**_ ^b^	22912.79	0.38	1.16	171.64	0.09	6.39E-06	7.61E-05
**σ**^**2**^_**g-p**_ ^b^	0.16	1.75E-08	2.70E-14	7.30E-07	4.03E-14	1	1
***Error*** ^b^	297633.70	0.49	0.96	158.62	0.08	63.18	47.54
**h**^**2**^ ^a^	0.86	0.74	0.30	0.88	0.85	0.80	0.66
**h**^**2**^ ^b^	0.13	0.61	0.71	0.52	0.63	1.01E-07	1.60E-06
**σ**^**2**^_**g**_ ^a^^,^^b^	36735.76	0.35	1.22E+00	95.54	0.08	2.15E+01	2.036137
**σ**^**2**^_**g-p**_ ^a^^,^^b^	3.80E-41	1.14E-16	4.58E-17	5.70E-16	2.49E-47	1.86E-08	1.00E+00
***Error*** ^a^^,^^b^	269451.6	0.77	12.74	196.67	0.11	52.01	49.57
**h**^**2**^ ^a^^,^^b^	0.35	0.60	0.28	0.46	0.73	0.44	0.07

YIELD: yield (Kg/ha); WGP: whole grain protein; SW: specific weight; gluten index, GI; initial agronomic score, IAS; sedimentation index, SDS; and yellow color, YC); *g*: genotype variance; *g-p*: significance test for genotype variance; *ge*: genotype-by-environment interaction variance; *ge-p*: significance test for genotype-by-environment interaction variance. NA: 'ge' couldn't be calculated due to data without any replications.

^**a**^ Durum wheat varieties.

^**b**^ Experimental durum wheat lines.

**Fig 1:**
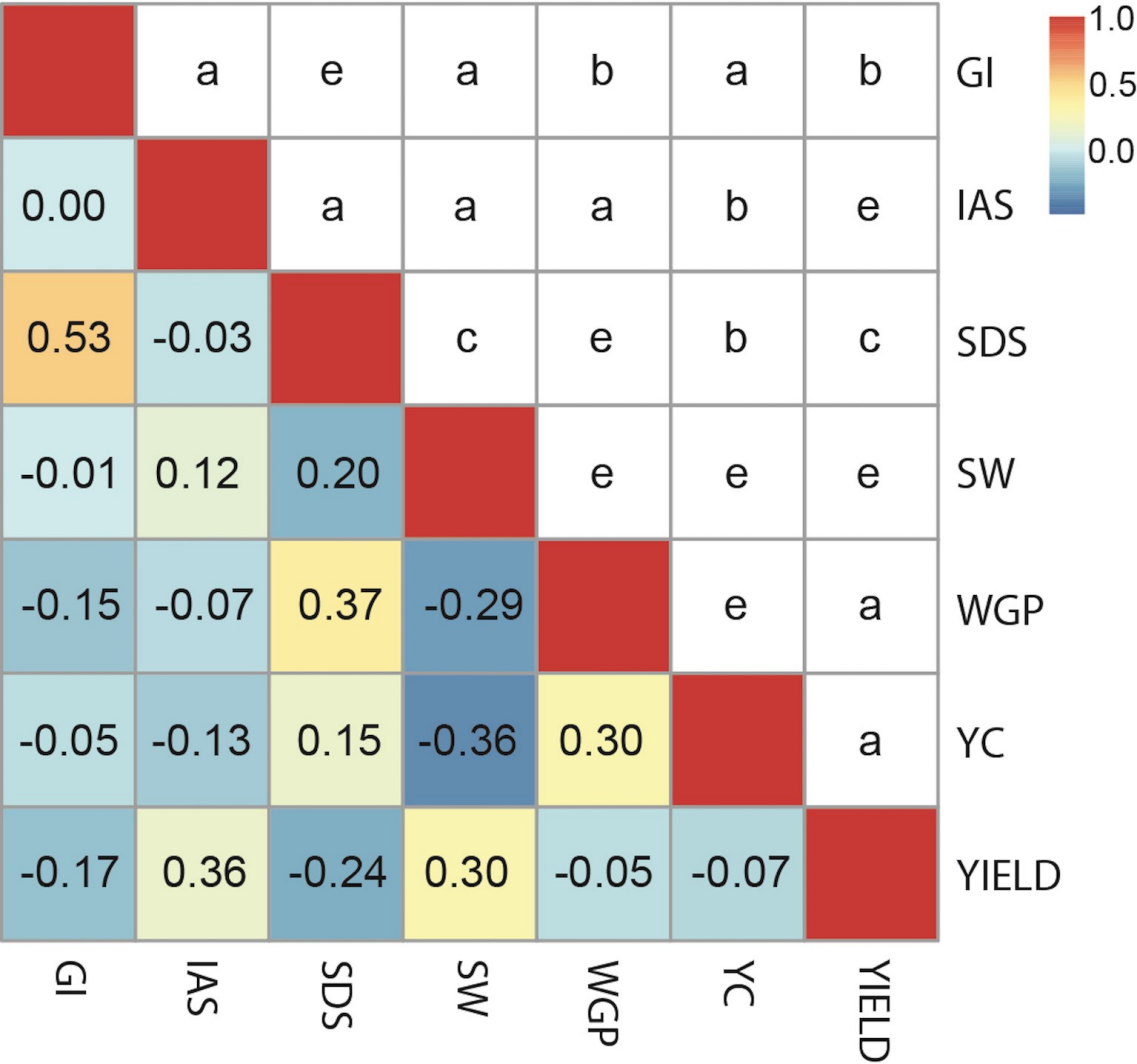
Phenotypic correlations found among assessed traits. GI: gluten index; IAS: initial agronomic score; SDS: sedimentation index (SDS); SW: specific weight; WGP: whole grain protein; YC: yellow colour; and YIELD: grain yield. Above, the range for *p-values* was indicated using a scale from “a” to “e” (a: represents *p-values* larger than 0.1; b: represents values between 0.1 and 0.01; c: represents values between 0.01 and 0.001; d: indicates values between 0.001 and 0.0001; e: for values between 0.0001 and 0.00001); below, correlations are shown using a colour scale (highest correlations in red, lowest correlations in blue).

The phenotypic correlation values presented a wide range. The highest value observed was r = 0.53 between GI and SDS, followed by SDS—WGP (r = 0.37), SW—YC (r = 0.36) and SW–YIELD, and also WGP—YC (both r = 0.30). SDS and YIELD showed an intermediate value of r = 0.24. The lowest values were found for GI-IAS, GI-SW, IAS-SDS, GI-YC, WGP-YIELD, IAS-WGP and YC-YIELD (ranging from 0 to 0.07) (Fig 1, S7 Table).

### DArT and SNP genotyping, principal coordinates and linkage disequilibrium analysis

A total of 5,711 SNPs and 14,979 DArT markers were mapped across the two constitutive genomes of durum wheat. In the case of SNP markers, 44% of the markers were located on the A genome and 56% on the B genome. The highest marker density was found in chromosomes 1B, 2B, 5B and 7A with a total of 558, 550, 512 and 496 markers, respectively. Chromosomes 4B and 5A showed the lowest number of located loci (217 and 231, respectively). For DArT markers, 41% of the markers were placed on the A genome and 59% on the B genome. The highest marker density was found in chromosomes 3B, 1B, 2B and 6B with a total of 1,593, 1,439, 1,427 and 1,416 loci, respectively. Chromosomes 4B and 5A contained the lowest number of loci (500 and 447, respectively) (Table 2).

**Table 2:** Distribution of 5,711 SNP and 14,979 DArT markers mapped across the two constitutive genomes (A, B) of durum wheat.

Chromosome	No. loci (SNPs)	No. loci (DArTs)	Total
1A	255	644	899
1B	558	1,439	1,997
2A	471	1,098	1,569
2B	550	1,427	1,977
3A	344	834	1,178
3B	475	1,593	2,068
4A	347	1,258	1,605
4B	217	500	717
5A	231	447	678
5B	512	1,217	1,729
6A	318	854	1,172
6B	409	1,416	1,825
7A	496	1,017	1,513
7B	409	1,235	1,644

**Fig 2:**
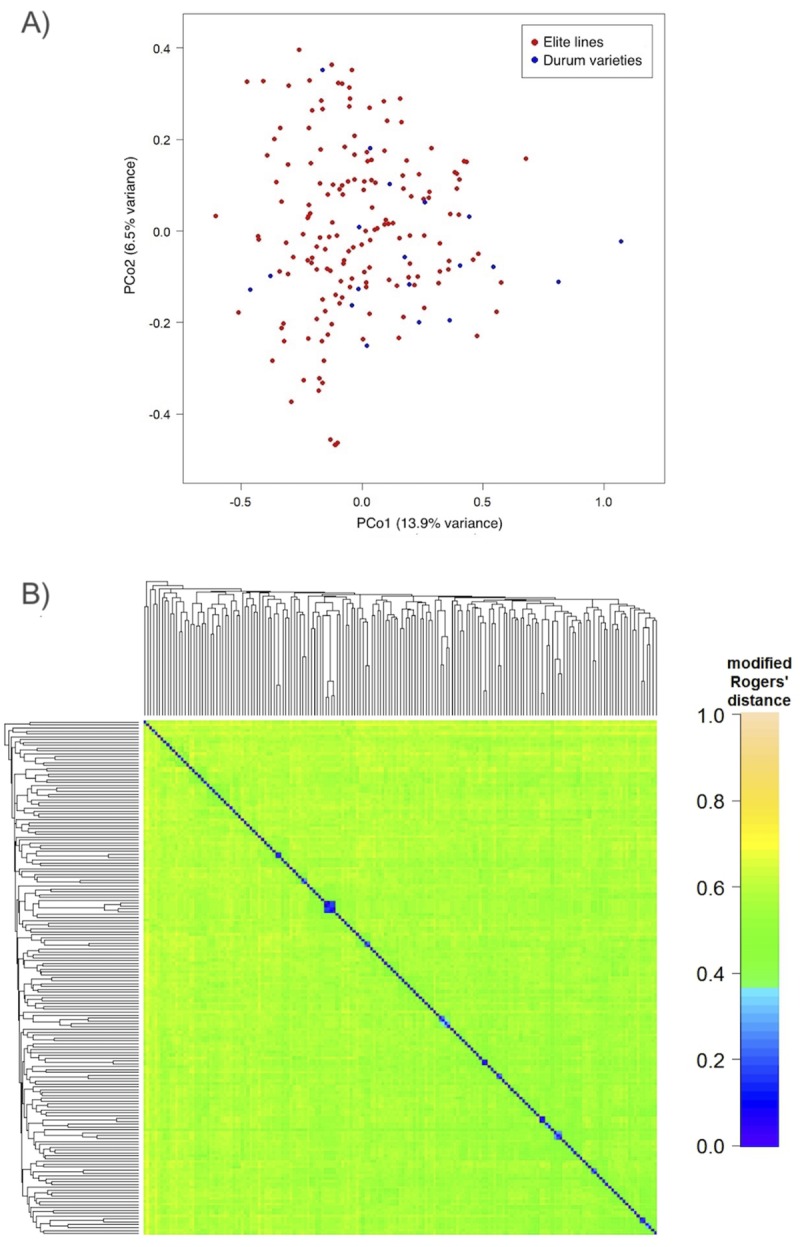
Population structure analysis. a) Principal Coordinates Analysis (PCoA) of the durum wheat panel assessed. The graph shows first versus second coordinates; b) Heatmap showing pairwise modified Roger’s distance among 179 lines genotyped by 5,649 SNP markers. Average linkage clustering was used to order the lines.

**Fig 3:**
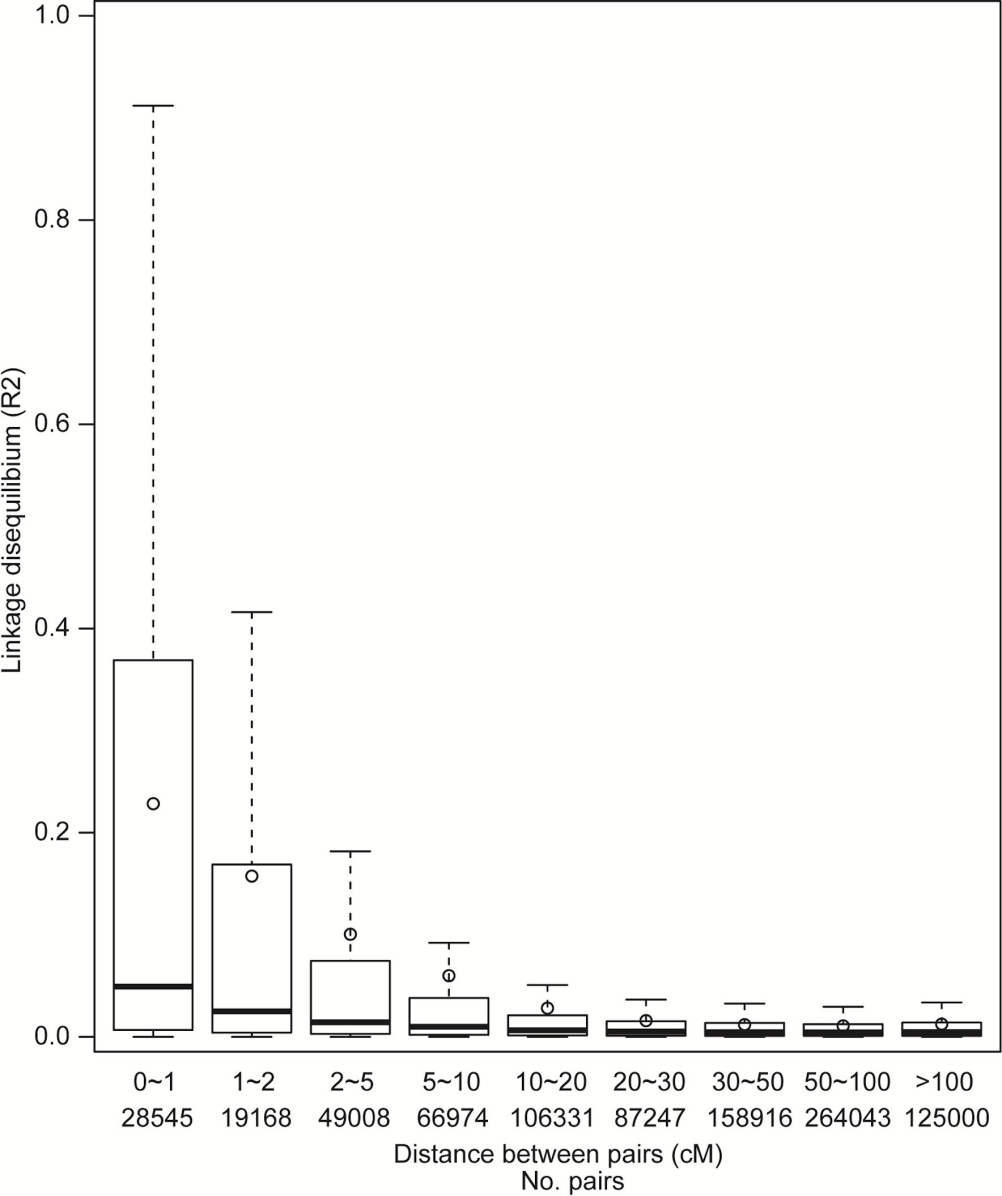
Linkage disequilibrium (LD) analysis of the line set. R^2^: correlation between a pair of loci; cM: centimorgan.

PCoA was applied to investigate the population structure in the line set (Fig 2A). The first and second principal coordinates accounted for 13.93% and 6.47% of the molecular variance, respectively. No significant genetic structure was detected. The heatmap plot for modified Roger’s distance was used to validate the result (Fig 2B). The PCos and eigenvalues obtained are shown in S8 and S9 Tables, respectively. As part of chromosome linkage disequilibrium (LD) assessment, pair-wise focusing on the mapped SNP markers was carried out. The R^2^ value between marker pairs fell below 0.2 at around 1 to 5cM (Fig 3).

### Marker-trait associations

**Fig 4:**
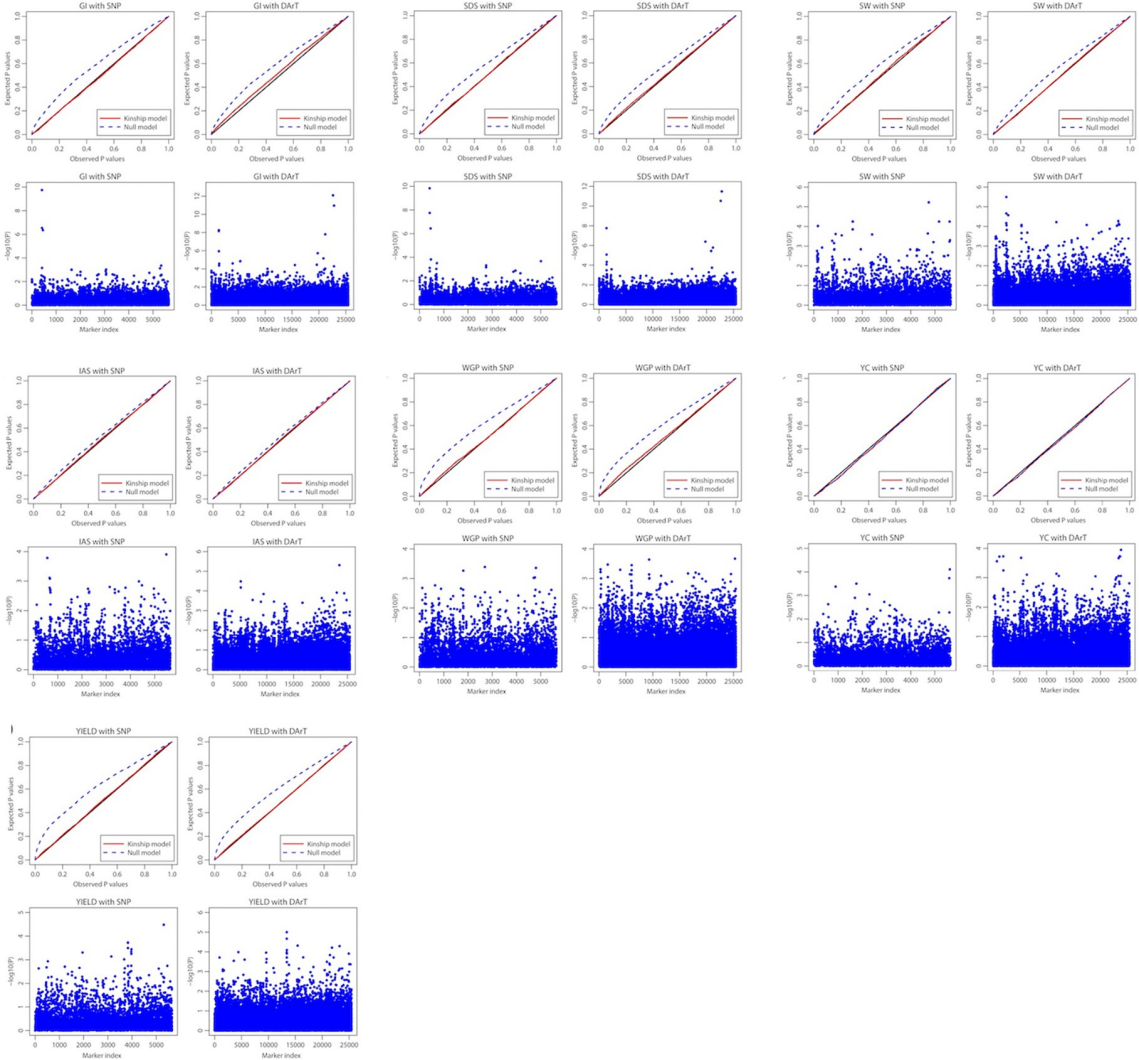
Quantile-quantile plots for the GWAS model and Manhattan plots for the assessed traits. (GI: gluten index; SDS: sedimentation index; SW: specific weight; IAS: initial agronomic score; WGP: whole grain protein; YC; yellow colour; and YIELD: grain yield). Expected and observed P values are shown on QQ-plots. Dotted blue lines represent the null model; red lines show the kinship model. Manhattan plots illustrate the marker index for each trait and the significance of the association test (as the negative logarithm of the P value).

Quantile-quantile plots were used and expected and observed log_10_ P-values were compared for the SNP and DArT marker datasets separately (Fig 4, S10 and S11 Tables). The correlation between the SNP and DArT kindship matrices (S4 and S5 Tables) was 0.938. As we had noted the absence of a pronounced population structure (Fig 2), we only fixed a group effect for the kinship model analysis (advanced lines vs. tester varieties), which improved the null model for most traits (Fig 4).

After analysis of the seven agronomic traits assessed, 37 MTAs were found for three quality traits (gluten index, GI; specific weight, SW; and sedimentation index, SDS) (Table 3). Twenty of the markers were found in association with GI, corresponding to 17 DArTs (7 unmapped) and 3 SNPs, located on chromosomes 1B, 2B, and 3A and accounting for 0.02 to 23.32% of the phenotypic variation. Ten markers were associated with SDS: 7 DArTs (4 unmapped) and 3 SNPs, all placed on chromosome 1B, which accounted for 0.06 to 16.14% of the phenotypic variation. Finally, one DArT and six SNPs (three of them unmapped and the rest located on chromosomes 1A, 2A and 3A) were associated to SW, accounting for 0.58 to 5.79% of the phenotypic variation (Table 3). The marker effects were within a 0.11–18.49 range (Table 3). Nine markers (8 associated to GI and 1 to SDS) showed the highest marker effects (7.3–18.49 range). Among the GI MTAs, marker *DArT1707*, placed on chromosome 1B, presented the highest additive effect value (18.49), followed by *DArT22904* and *DArT26318*, both unmapped, with effects of 11.52 and 10.50, respectively. We can also highlight marker effects for *DArT1762* and *DArT6596*, placed on chromosomes 1B and 3A, with values of 9.85 and 9.52, respectively. Linked to SDS, the markers *DArT26104* (unmapped) and *DArT24559*, placed on chromosome 1A, showed effects of 7.37 and 5.46, respectively. Finally, for SW, the marker effects had a narrower range from 0.1 (*DArT2892*) to 1.62 (*SNP2318*). Two major associations were detected, one for GI (marker *DArT26104*; R^2^ = 23.32%) and one for SDS (marker *DArT26318*; R^2^ = 16.14%), based on Flint-Garcia et al [5], who described ‘major QTLs’ as those characterized by 10% R^2^ detected in AM analysis.

**Table 3:** Marker-trait associations found for quality traits.

Trait	Marker	Chr	Pos. (cM)	R^2^ (%)	Marker effect
SW	SNP219	1A	205.3	3.3	0.616
SW	DArT2892	2A	63.6	2.95	0.106
SW	SNP2318	3A	69.6	0.58	1.617
SW	SNP2323	3A	70.9	5.79	0.674
SW	SNP7042	-	-	0	0.357
SW	SNP8003	-	-	-	-
SW	SNP9057	-	-	-	-
	*Residuals*			87.37	
GI	DArT4742	2B	78.1	5.49	-7.504
GI	DArT6596	3A	125.2	1.86	9.521
GI	DArT6585	3A	125.2	0.02	-1.253
GI	DArT6586	3A	125.2	-	-
GI	DArT1707	1B	130.4	0.36	-18.498
GI	DArT24559	1B	130.4	0.44	-4.254
GI	SNP614	1B	136.0	0.02	-2.006
GI	DArT1740	1B	136.0	0.14	-0.648
GI	SNP616	1B	137.2	2.01	-8.838
GI	DArT1744	1B	138.4	2.06	-9.491
GI	DArT1762	1B	141.2	0.44	-9.849
GI	DArT1806	1B	146.1	1.55	4.076
GI	SNP670	1B	146.7	0.43	-2.120
GI	DArT26104	-	-	23.32	-4.122
GI	DArT26318	-	-	0.94	-10.499
GI	DArT23081	-	-	1.45	3.690
GI	DArT24191	-	-	0.41	-4,394
GI	DArT22904	-	-	4.97	-11.522
GI	DArT18751	-	-	1.69	4.672
GI	DArT26304	-	-	0.04	-1.406
	*Residuals*			52.36	
SDS	DArT1707	1B	130.4	0.53	-4,341
SDS	SNP614	1B	136.0	0.44	-0.206
SDS	SNP616	1B	137.2	0.06	-2,289
SDS	SNP670	1B	146.7	1.73	-3,431
SDS	DArT26318	-	-	16.14	-1,209
SDS	DArT26104	-	-	2.65	-7,371
SDS	DArT23081	-	-	0.42	2,569
SDS	DArT24559	1A	-	0.16	-5.46
SDS	DArT24191	-	-	0.32	-1,025
SDS	DArT1744	-	-	0.75	4,701
	*Residuals*			76.78	

SW: specific weight; GI: gluten index; SDS: sedimentation index; R^2^: percentage of phenotypic variation explained by the marker; cM: centimorgan.

### Candidate genes

**Fig 5:**
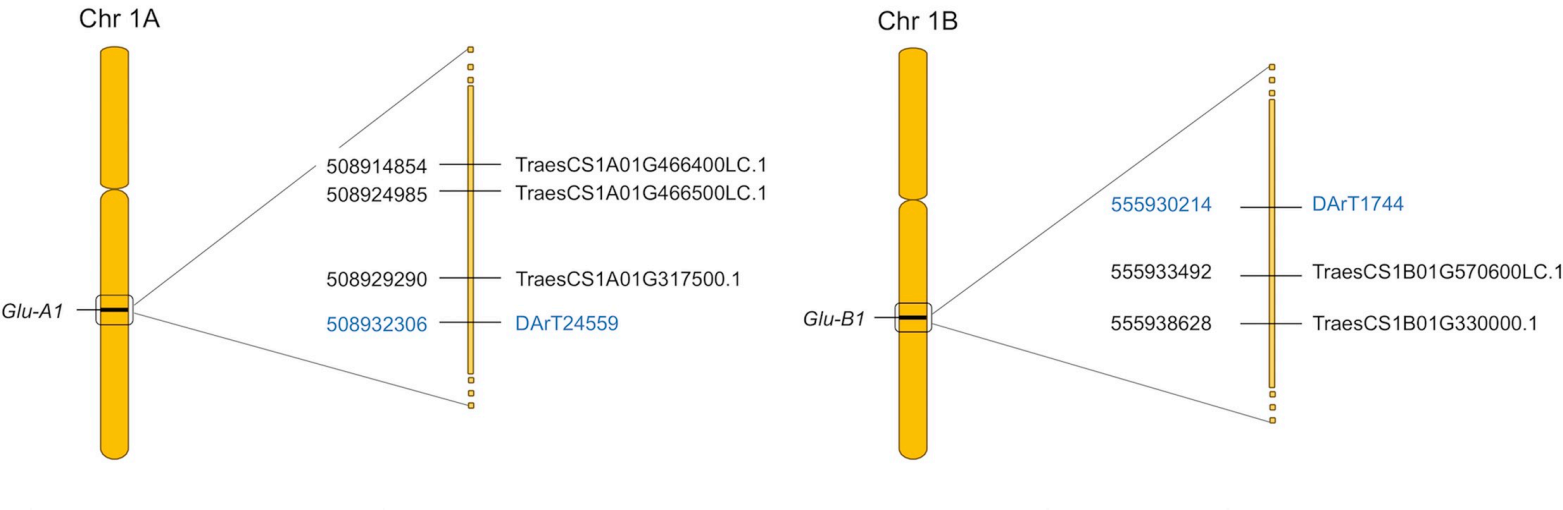
**Candidate genes** and related markers located on chromosomes 1A and 1B.

BLAST analyses of DArT and SNP sequences on the Enssemble genome browser for the wheat genome (https://plants.ensembl.org/Triticum_aestivum/Info/Index) showed that two DArT markers were related to some important proteins with nutrient’s reservoir activity (Fig 5, Table 4). The marker *DArT1744* (located in chromosome 1BL) was associated with GI, and corresponds to the *Glu-B1* locus [51]. It is very closed to two high molecular weight (HMW) subunit genes: *TraesCS1B01G570600LC*.*1* (3278kb from the marker) encoding a Glu1B y-type HWM glutenin subunit; and *TraesCS1B01G330000*.*1* (8414kb), encoding a Globulin 1 protein. The marker *DArT24559* (located in chromosome 1AL) was associated to SDS, and corresponds to the *Glu-A1* locus. It is located closed to three HMW subunit genes: *TraesCS1A01G317500*.*1* (-3016kb from the marker) encoding a Globulin 1 protein; *TraesCS1A01G466400LC*.*1* (-17452kb) encoding a Glu1Ay; and *TraesCS1A01G466500LC*.*1* (-7321kb) encoding a Glu1Ay protein. Differential expression analyses highlighted two of these high confidence genes, *TraesCS1B01G330000*.*1* in chromosome 1BL, and *TraesCS1A01G317500*.*1* in chromosome 1AL (Fig 5), which are differentially expressed under different drought stress conditions (Sl Fig, [52, 53]).

**Table 4:** Genes located in the proximity of markers *DArT1744* and *DArT24559* (within a +-50kb window).

Marker	Chr	Position (kb)	Identity	Adjacent *T*. *Aestivum* gene	Distance	Protein	Gene id	Description	Predicted function
DArT1744	1B	555930214 555930282	100	TraesCS1B01G570600LC.1	3278	W6AWK6_WHEAT	*Glu y-type*	High molecular weight subunit	Nutrient reservoir subunit
				TraesCS1B01G330000.1	8414	Q0Q5D9_WHEAT	*Globulin 1*	High molecular weight subunit	IgE binding
				TraesCS1B01G570400LC.1	-34468			DNA-binding protein with MIZ/SP-RING zinc finger. PHD-finger and SAP domain-containing protein	RNaseH-like_sf
				TraesCS1B01G329900.1	-21416	A0A341P5G3_WHEAT		Werner Syndrome-like exonuclease	RNaseH-like_sf
				TraesCS1B01G570500LC.1	-20301			Serine/threonine-protein phosphatase 2A 55 kDa regulatory subunit B alpha isoform	
				TraesCS1B01G570700LC.1	3400			Imidazole glycerol phosphate synthase subunit HisF	
				TraesCS1B01G330100.1	18578	W5A1N6		Receptor kinase	Kinase-like_dom_sf
DArT24559	1A	508932306 508932238	94.203	TraesCS1A01G466400LC.1	-17452	A0A2U8T924_WHEAT	*Glu-1Ay*	High molecular weight subunit	Nutrient reservoir subunit
				TraesCS1A01G466500LC.1	-7321	A0A2U8T924_WHEAT	*Glu-1Ay*	High molecular weight subunit	Nutrient reservoir subunit
				TraesCS1A01G317500.1	-3016	Q0Q5E3_WHEAT	*Globulin 1*	High molecular weight subunit	IgE binding
				TraesCS1A01G466300LC.1	-29283			DNA topoisomerase 2-binding protein 1-A	
				TraesCS1A01G466600LC.1	-3693			Ribonuclease H-like superfamily protein	
				TraesCS1A01G466700LC.1	-219			Leucine-rich repeat receptor-like protein kinase family protein	
				TraesCS1A01G317600.1	12397	A0A341NRU4		Retrovirus-related Pol polyprotein from transposon TNT 1–94	
				TraesCS1A01G466800LC.1	28336	T1NHT9		Transposase	
				TraesCS1A01G317700.1	29723	A0A341NQ24		ARM repeat superfamily protein	
				TraesCS1A01G466900LC.1	29780			Immunoglobulin G-binding protein A	
				TraesCS1A01G467000LC.1	31167			Retrovirus-related Pol polyprotein from transposon TNT 1–94	
				TraesCS1A01G467100LC.1	32907	A3FKK9		Receptor protein kinase	Kinase-like_dom_sf; Xa21-like protein (T. Turgidum)

### Genome-wide prediction analysis

**Fig 6:**
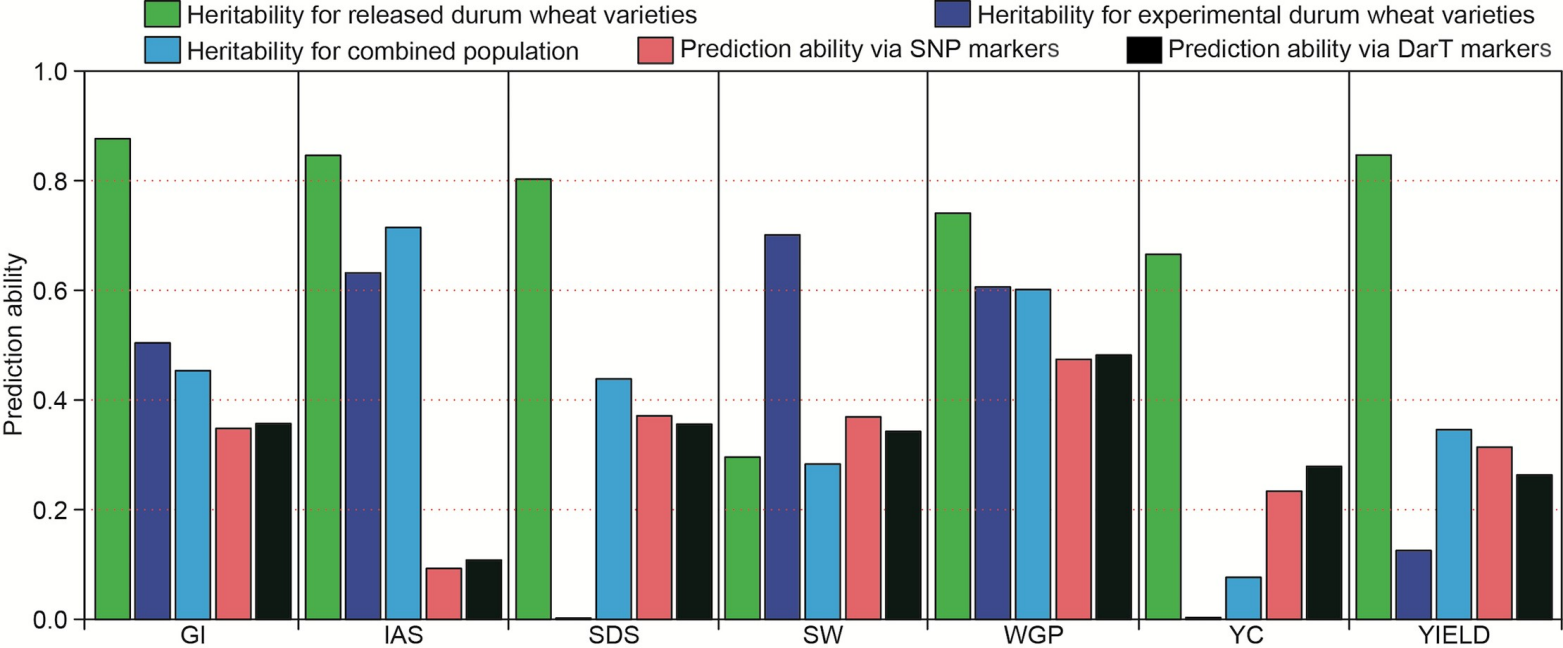
Genomic selection and heritability. a) Genomic selection accuracies for 179 lines using SNP and DArT markers for the assessed traits. b) Relationship between prediction ability and heritability. GI: gluten index; IAS: initial agronomic score; SDS: sedimentation index; SW: specific weight; WGP: whole grain protein; YC: yellow colour; and YIELD: grain yield.

Genome-wide prediction ability was calculated and was represented for the seven traits assessed in the 179 genotypes panel, using 16,383 DArT and 5,649 SNP markers (Fig 6). There were slight differences between both marker types in their prediction ability for the same trait, ranging from 0.01 to 0.05 (Table 5). The highest prediction accuracy was found for WGP (r = 0.482 using DArTs and r = 0.474 with SNPs), followed by SDS (r = 0.371 using SNPs), while the lowest values were obtained for IAS (r = 0.108 with DArTs and r = 0.093 using SNPs). Four of the traits showed higher prediction values using DArT markers (GI, IAS, WGP and YC) and three traits using SNP markers (YIELD, SDS and SW).

**Table 5:** Genomic selection (GS) prediction ability results for the assessed traits using DArT and SNP markers.

Traits	DArTs	SNPs
GI	0.357	0.348
IAS	0.108	0.093
SDS	0.356	0.371
SW	0.343	0.369
WGP	0.482	0.474
YC	0.279	0.234
YIELD	0.263	0.314

GI: gluten index; IAS: initial agronomic score; SDS: sedimentation index; SW: specific weight; WGP: whole grain protein; YC: yellow colour; and YIELD: grain yield.

## Discussion

Field experiments for the assessment of yield and quality traits under rainfed conditions were carried out at five sites in Southern Spain. These Mediterranean environments present unpredictable water deficit and heat stress during the final stages of wheat development, affecting the mentioned traits. A strong effect of maximum temperatures on yield was observed at final stage (S2A Fig), while thermal sum (GDD) presented a moderate to minor effect (S2B Fig).

Yield is greatly influenced by both environmental conditions and genotype [54, 55], resulting in low plot-based heritabilities under water stress conditions [56, 57]. Previous studies performed in durum wheat, showed variations in yield heritability caused by differences in environmental conditions [55, 57–60]. In line with this, our results showed low plot-based heritability for yield (h^2^ = 0.13) over the different locations and years of assessment. This is in agreement with Gonzalez-Ribot et al. [57], who obtained a low plot-based heritability for yield (h^2^ = 0.24), in unrelated high-yield durum lines grown under water stress in Mediterranean environments.

As previous studies highlighted [61–64], yield is negatively correlated to protein content (WGP) (r = -0.29) (S6 Table); and an increment in protein content results in reductions in final yield [65]. It has been highlighted that there is no genetic basis for this negative correlation, since strong environmental and physiological interactions are in charge [66]. Nevertheless, Groos et al. [63] showed that this negative correlation could be due to a close genetic relation or contrary effects produced by environmental conditions in both traits.

Blanco et al. [67] emphasised that yield and protein content are managed by a complicated genetic system which is influenced by environmental conditions and agricultural practices. As result of the environmental influence, differences in final YIELD and WGP were observed between locations and years (S12 Table). Variance component analyses showed that the effect of genotype-by-environment interactions was far higher for WGP than in the case of YIELD (Table 1). These results agree with previous studies which reported that protein content is strongly influenced by environmental conditions [68, 69]. Protein content usually presents high heritability values [70, 71]. In this study, a moderate to high value was obtained for WGP heritability (h^2^ = 0.62) in comparison with previous studies [67, 72] reporting heritabilities in the 0.54–0.78 range for durum wheat recombinant inbred lines (RILs) grown at several Mediterranean environments.

Gluten strength (GStr) is a highly significant trait in durum wheat [73], in direct relation to GI and SDS, which are considered a measure of GStr [39, 74]. Both traits have been described as highly inheritable [74] and show a strong correlation [32, 73, 74]. In agreement with these findings, our results showed high heritability values for GI (h^2^ = 0.88) and SDS (h^2^ = 0.80), and also a positive correlation between them (r = 0.53).

The genome-wide association analysis is becoming a popular approach to dissect the genetic base of complex traits in durum wheat. Previous AM and QTL mapping studies found QTLs involved in quality traits in most of chromosomes [34, 72, 75–79]. In this work, the AM approach taken over the years and different locations, resulted in 37 significant markers associated with three important quality traits (gluten index, sedimentation index and specific weight) in known and novel genomic regions (Table 3). Most of the markers associated with GI were located on chromosome 1B (0.02–2.06% of phenotypic variation), where major genomic regions for gluten strength and several genes related to endosperm proteins as gliadin and glutenin subunits are located [80–83]. The remaining MTAs for GI were located in chromosomes 2B (5.49%) and 3A (0.02–1.86%). In line with these results, previous studies carried out in durum wheat, under similar limiting conditions, found DArT markers in association with GStr in several chromosomes, including 1B (0.07–0.16% phenotypic variation) and 3A (0.04–0.06%) [84].

Markers found in association with SDS were all located on chromosome 1B (0.06–16.14% of phenotypic variation), consistent with previous studies across environments and conditions, which used different marker types and populations [78, 79, 85] (RILS, F_2:7_, F_9_ or double haploids, respectively). Bread wheat MTA studies also found major QTLs associated with SDS in this chromosome [76, 86].

Finally, novel MTAs for SW were found on chromosomes 1A, 2A and 3A (0.58 to 5.79% of phenotypic variation). Studies in durum and bread wheat, carried out in a wide range of environments and conditions, placed markers in association with this trait in several other chromosomes [35, 75, 84]. A recent study in durum wheat landraces, performed in Northern Spain under rainfed conditions [87] found significant DArT markers associated with SW in several chromosomes, including 3A (0.07–0.09% of variation), but in a different position.

The relationship between durum wheat gluten strength and HMW- glutenins is well known and controlled by major loci [51]. While we did not observed MTAs for the *Gli-B1* locus, consistent with the previous selection carried out for the favourable γ-gliadin 42 allele in this elite material, we could detect MTAs for the *Glu-B1* [78, 87, 88] and *Glu-A1* [88, 89] loci (markers *DArT1744* and *DArT24559*). By blasting both markers, we have precisely mapped the *Glu-B1* and *Glu-A1* loci on the wheat reference genome (IWGSC 2018) and proposed the corresponding candidate genes among the gene models annotated as HMW subunits (Table 4). In agreement with our results, several major and meta QTLs for quality under drought stress reported the *Glu-A1* locus [89]. The marker *DArT1744* (chromosome 1BL) associated with GI, was found close to the gene models *TraesCS1B01G570600LC*.*1* and *TraesCS1B01G330000*.*1*, encoding for HMW glutenin subunits (a Glu1B y-type and a Globulin 1 proteins respectively). The locus *Glu-B1* was previously located within a meta-QTL (MQTL6) which contains several QTLs for yield components and gluten strength [78, 88, 89]. The marker *DArT24559* (chromosome 1AL), in association with SDS, was located within MQTL6 [78, 88, 89] in the proximity to the gene models (*TraesCS1A01G466400LC*.*1*, *TraesCS1A01G466500LC*.*1* and *TraesCS1A01G3175 00*.*1*), also encoding HMW subunits (Glu1A y-type and a Globulin 1). These novel markers and candidate genes located on the RefSeqv1 wheat genome reference [78, 88, 89] for the known *Glu-B1* and *Glu-A1* loci are new resources for durum wheat breeding and support the potential of the GWAs approach.

The use of models focused on genomic prediction in wheat breeding programs reduces the breeding cycle, giving an increase in genetic gains. Nevertheless, genomic prediction studies taking into account the genotype-by-environment (GxE) interactions are still reduced on durum wheat [33]. In this work, we applied the genomic selection (GS) approach to elite and durum wheat varieties, phenotyped under rainfed conditions (Fig 6A, Table 5). The highest GS prediction accuracy was found for WGP (r = 0.482 using DArTs and r = 0.474 using SNPs) which could be considered to fall within a similar range as previous reported by Fiedler et al. [75] (r = 0.56) using more lines (1184 breeding durum wheats (F_4:7_)) and several conditions; or Bentley et al. [90] (r = 0.66; r = 0.58), who analysed 376 winter wheat varieties, grown in field experiments across different environments for a long period, using DArT markers.

Prediction accuracy values for YIELD (r = 0.263 with DArTs and 0.314 with SNPs) are similar to those reported by Sukumaran et al. [33] (from 0.20 to 0.40) applying several prediction models and basic cross-validation strategies for the assessment of durum wheats grown under different stresses, as drought and heat conditions. Yield prediction accuracies were lower than for WGP (r = 0.482 with DArTs and 0.474 with SNPs). These results contrast with those obtained by Bentley et al. [90] for winter wheats, who showed more similar GS prediction values for both of these traits, with yield results slightly better than those of protein content. Differences found between these studies could reside in the fact that both traits are heavily influenced by environment conditions and genotype-by-environment interactions [54, 55, 91].

Our GS analysis showed promising results which support its use in current plant breeding programs. The prediction accuracies obtained were fairly similar for the two marker systems used: DArTs and SNPs (Table 5), despite the fact that the number of DArTs almost tripled that of the SNPs (16,383 vs 5,649 respectively). These results, leveraged with the corresponding marker prices, could be useful when selecting future marker systems.

## Conclusion

Association mapping and genomic selection approaches were applied using the same genotyped and phenotyped collection of experimental lines and varieties of durum wheat. The main aim of AM was to detect specific loci on the wheat genome which were directly related with phenotypic character variations, while GS uses statistical models to predict genomic values for the assessed lines.

The AM approach revealed interesting marker-trait associations over the years and in the different environments for three quality traits (gluten index (GI), sedimentation index (SDS) and specific weight (SW)), which is of great importance for the final durum wheat product, and presented a wide range of effects in the phenotype expression. Most associated DartSeq and SNP markers were mapped to the A and B bread wheat sub-genomes using the available closely-related bread wheat reference IWGSC RefSeqv1. The application of GS was successful for most of the traits in the breeding materials analysed and showed promising results, especially for quality traits such as grain protein content or those in which MTAs were found (SDS, SW and GI). GS showed promising results which support its use in current plant breeding programmes. These results can be used in current plant breeding programmes for key quality traits in durum wheat under Mediterranean rainfed conditions with a limited water supply.

## Supporting information

S1 Fig **Analysis of candidate genes found in *Glu-A1* and *Glu-B1* loci in chromosomes 1A and 1B.** Differentially expression was indicated for each gene: SFS—severe stress field conditions; MFS—mild field stress conditions; P1h - osmotic stress as polyethylene glycol (PEG) 1hour; P6h - osmotic stress PEG 6hours.
(TIFF)

S2 Fig Relationship between yield and temperature. a) Relation between yield and maximum temperature mean (°C) by location and year for durum wheat varieties for final stages; b) Relation between yield and thermal sum from 1^st^ April to 30^th^ June. (YIELD: mean values by place and year for released lines (Kg/ha); Tmax: maximum temperature (°C); GDD: Growing Degree Days, thermal sum using 4°C as base temperature).
(TIFF)

S1 Table List of durum wheat lines assessed. (XLSX)

S2 Table Meteorological information collected from agroclimatic stations (Junta de Andalucia) (https://www.juntadeandalucia.es/agriculturaypesca/ifapa/ria/servlet/ FrontController). Mean, maximum and minimum values for temperature (maximum temperature (Tmax), average temperature (Tmed) and minimum temperature (Tmin)) (°C), rainfall (Pp) (mm) and evapotranspiration (Eto) (mm/day) for the five locations assessed. Daily values are also include in separated sheets for each location.
(XLSX)

S3 Table Site location and agronomical details. (XLSX)

S4 Table Kindship matrix for DArT markers. (XLSX)

S5 Table Kindship matrix for SNP markers. (XLSX)

S6 Table Descriptive statistics of each trait in each environment with key quantiles. (XLSX)

S7 Table Matrix of correlations between assessed traits across years and environments (yield (Kg/ha), YIELD; yellow colour, YC; whole grain protein (%), WGP; specific weight (g), SW; sedimentation index (cm3), SDS; initial agronomic score, IAS; and gluten index (%), GI). (XLSX)

S8 Table Projection of each genotype assessed on all the PCos. (XLSX)

S9 Table Eigenvalues from the PCo analysis. (XLSX)

S10 Table BLUP analysis results using DArT markers for assessed traits (gluten index, GI; initial agronomic score, IAS; sedimentation index, SDS; specific weight, SW; whole grain protein, WGP; yellow colour, YC; yield, YIELD). (XLSX)

S11 Table BLUP analysis results using SNP markers for assessed traits (gluten index, GI; initial agronomic score, IAS; sedimentation index, SDS; specific weight, SW; whole grain protein, WGP; yellow colour, YC; yield, YIELD). (XLSX)

S12 Table Accession mean values and standard deviation for assessed traits by year and location. (IAS, initial agronomic score; WGP, whole grain protein (%); SW, specific weight (g); YC, yellow colour; GI, gluten index (%); SDS, sedimentation index (cm3); and YIELD, yield (Kg/ha)). Means were calculated for three replications of the durum varieties at the five locations, and one plot per genotype for experimental lines at two sites.
(XLSX)
