# Supplementary figures and images for: Genetic dissection of agronomic and quality traits based on association mapping and genomic selection approaches in durum wheat grown in Southern Spain
Source: PLoS One. 2019 Feb 27;14(2):e0211718. doi: 10.1371/journal.pone.0211718 (PMC6392243; doi:10.1371/journal.pone.0211718)

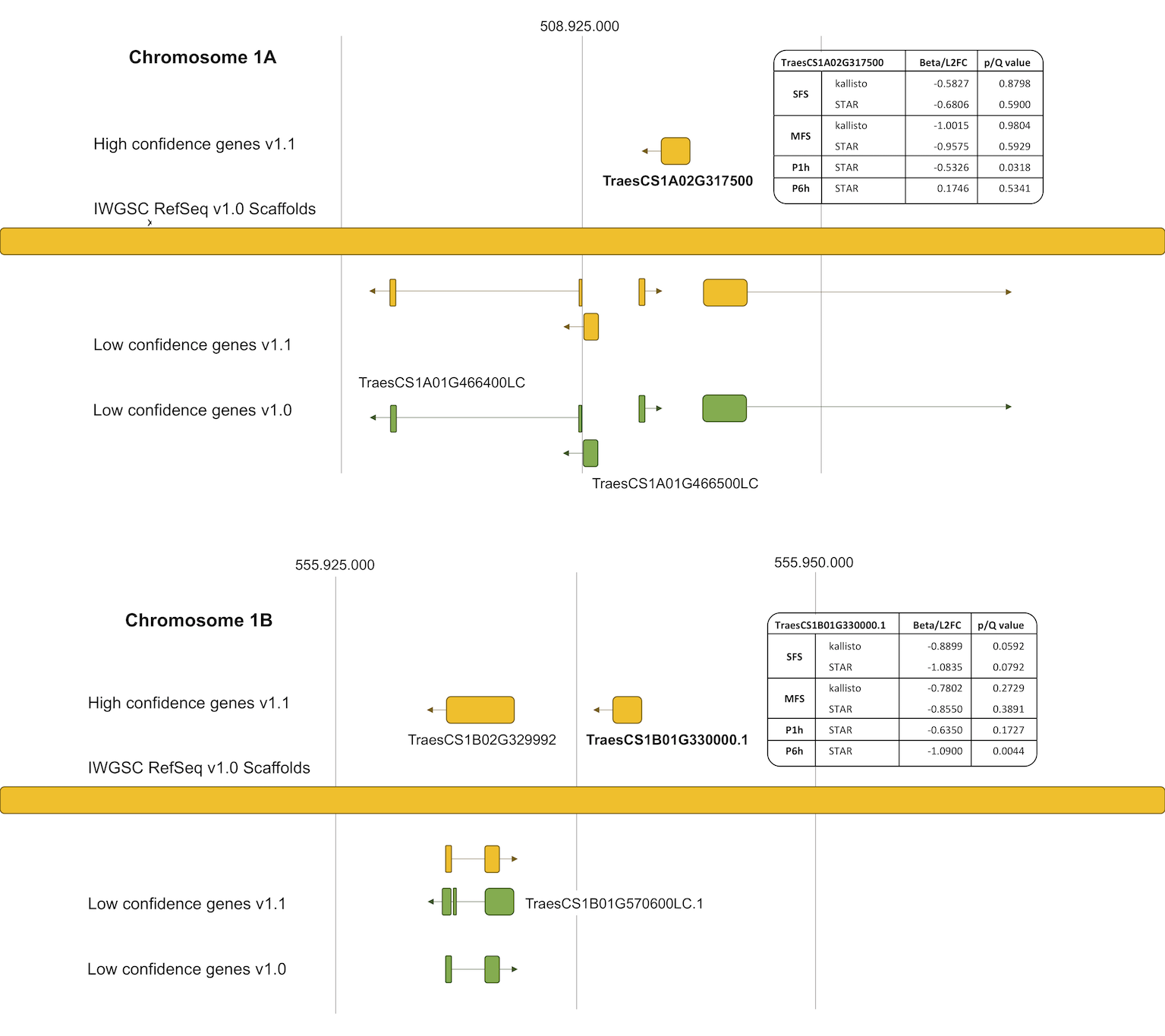

Supplement: S1 Fig — Analysis of candidate genes found in Glu-A1 and Glu-B1 loci in chromosomes 1A and 1B. Differentially expression was indicated for each gene: SFS—severe stress field conditions; MFS—mild field stress conditions; P1h - osmotic stress as polyethylene glycol (PEG) 1hour; P6h - osmotic stress PEG 6hours. (TIFF) [file pone.0211718.s001.tiff]

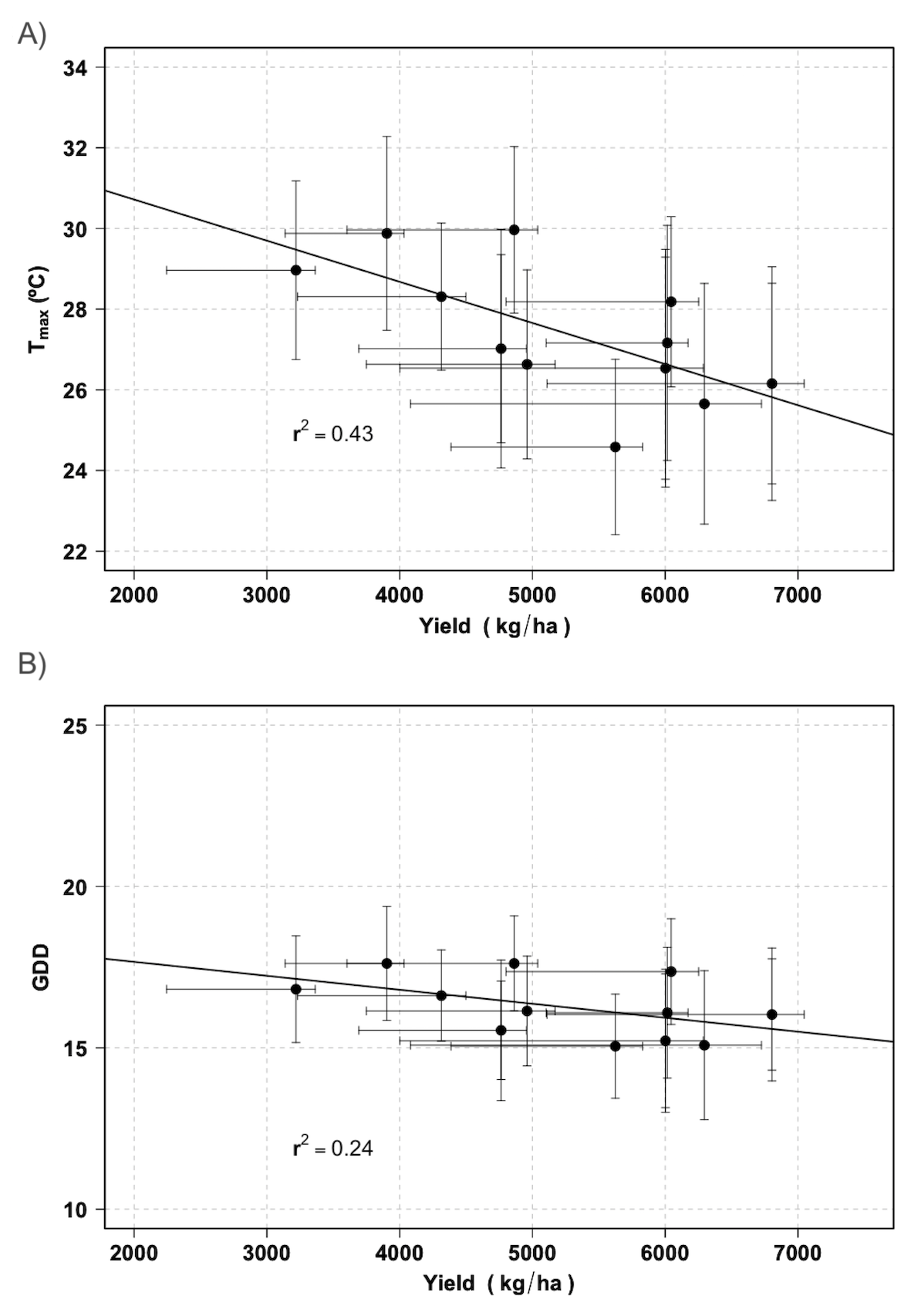

Supplement: S2 Fig — a) Relation between yield and maximum temperature mean (°C) by location and year for durum wheat varieties for final stages; b) Relation between yield and thermal sum from 1st April to 30th June. (YIELD: mean values by place and year for released lines (Kg/ha); Tmax: maximum temperature (°C); GDD: Growing Degree Days, thermal sum using 4°C as base temperature). (TIFF) [file pone.0211718.s002.tiff]
